# 

**Published:** 1875-12

**Authors:** 


### Contents of December Number.

### ORIGINAL.

ART. I.—On the use of Warm and Hot Water in Surgery. By Frederick E. Hyde, M. D., .......... 161

ART. II.—Chronic Dislocations of the Shoulder. By W. W. Miner, M. D. 181

ART. III.—Abstract of the Procedings of the Buffalo Medical Association, Nov. 2d, 1875, .......... 191

### EDITORIAL DEPARTMENT.

New Year's, .......... 200

